# AI-Empowered Mechanomedicine for Cancer-Related Lymphedema

**DOI:** 10.34133/research.1383

**Published:** 2026-08-04

**Authors:** Zhe Liu, Oscar Gonzalez, Minli You, Feng Xu, Ting Wen

**Affiliations:** ^1^ Department of Rehabilitation Medicine, The First Affiliated Hospital of Xi’an Jiaotong University, Xi’an 710061, P.R. China.; ^2^ Bioinspired Engineering and Biomechanics Center (BEBC), Xi’an Jiaotong University, Xi’an 710049, P.R. China.; ^3^ The Key Laboratory of Biomedical Information Engineering of Ministry of Education, School of Life Science and Technology, Xi’an Jiaotong University, Xi’an 710049, P.R. China.; ^4^ Hainan General Hospital, Hainan Affiliated Hospital of Hainan Medical University, Haikou 570311, P.R. China.

## Abstract

Cancer-related lymphedema is a chronic progressive side effect of cancer treatments followed by lymph node dissection or radiotherapy. Clinicians often identify lymphedema through limb swelling, while the disease begins earlier than that. Lymphatic injury is the original cause, where elevated interstitial fluid pressure and distorted tissue mechanics will lead to immune activation and fibrofatty remodeling. Recently, mechanobiology studies have deepened our understanding by linking lymph stasis to T helper 2/transforming growth factor β signaling, fibroblast mechanotransduction, and YAP/TAZ activity that together lock tissues into a stiff, poorly draining state. Simultaneously, emerging artificial intelligence (AI) in the field are being explored, from proof-of-concept image classification to much more diagnostic models that integrate elastography, indocyanine green lymphography, radiomics, clinical variables, and wearable signals to detect preclinical mechanical signatures and predict risk. These advances are driving the development of promising mechanomedical approaches, such as adaptive compression systems, AI-assisted plans for lymphatic reconstruction, anti-fibrotic strategies, and lymphangiogenic regeneration, although most remain at preclinical or early clinical feasibility stages of translation. We discuss the strength of current evidence, challenges for clinical translation, and standards for reporting. We propose that cancer-related lymphedema can be understood as a measurable and targetable mechano-immune-fibrotic disease, for which AI may eventually support earlier diagnosis, risk prediction, and personalized mechanotherapy.

## Introduction: Mechanical Cues Are Missing Hallmarks in Cancer-Related Lymphedema

Secondary lymphedema is a chronic condition characterized by limb swelling, fibrosis, and immune dysfunction. It often appears after cancer treatments like lymph node dissection or radiation and can severely impair patients’ quality of life [1,2]. As an emerging area of mechanomedicine, cancer-related lymphedema is closely related with mechanical cues in body tissues, which are critical regulators of the disease’s progression and targets for therapy. Historically, medical research has focused more on biochemical factors rather than mechanical ones. However, mechanical cues are starting to be recognized as important drivers of diseases ranging from cancer to fibrosis [3,4]. In the case of cancer-related lymphedema, mechanical cues like excess lymph fluid or tissue stretching and scarring play a crucial role in driving the disease’s development, yet they often receive insufficient attention in clinical practice.

Indeed, there exists a huge gap between recent mechanobiology insights and their translation to lymphedema patient care. Over the past few years, advances in biomechanics and mechanobiology have revealed that abnormal mechanical cues can trigger inflammation and fibrosis and impair regeneration of lymphedematous tissues [5,6]. Still, current treatments for lymphedema focus predominantly on symptoms rather than the underlying mechanisms causing them. Clinicians do not routinely check properties like tissue stiffness or lymphatic fluid dynamics, missing opportunities for early diagnosis and targeted therapy. As a result, many patients face long delays in their diagnosis, allowing lymphedema to advance into its irreversible stages [7]. For instance, one study recently found that it can take up to 10 years on average for lower-limb lymphedema to get diagnosed, due to its subtle and overlapping symptoms [8]. During such delays, ongoing mechanical stress from buildup creates a vicious cycle of inflammation and fibrosis that become harder to reverse.

An interdisciplinary approach is needed to bridge this gap. Mechanomedicine for lymphedema involves both understanding the mechanobiological foundations of the disease and developing technologies to monitor and adjust mechanical cues in patients. For that, artificial intelligence (AI) emerges as a powerful enabler. Current AI tools can analyze complex biomedical data, from imaging of tissue biomechanics to wearable sensor readings, detecting patterns invisible to the naked eye [9,10]. Thus, AI can empower mechanomedicine of lymphedema by (a) enhancing the diagnosis through recognition of mechanical biomarkers (e.g., subtle skin thickening or stiffness changes) and (b) guiding therapeutic decisions by predicting which interventions (compression, surgery, regenerative therapy, etc.) would best restore normal mechanics in each patient. This partnership between AI and mechanomedicine holds a great promise to transform lymphedema care from a reactive paradigm to a much more proactive and personalized one.

Recently, we witnessed that increasing studies have been reported about the AI applications in lymphedema risk prediction, imaging analysis, longitudinal monitoring, and treatment planning, suggesting that AI-empowered mechanomedicine may represent an important future direction for cancer-related lymphedema care. Thus, it is timely and significant to synthesize recent advances in understanding and utilizing mechanical cues related to cancer-related lymphedema. In this review, we find that cancer-related lymphedema is sustained by a self-reinforcing mechano-immune-fibrotic loop, and that AI makes this loop measurable, predictable, and therapeutically actionable. Our analysis begins exploring the mechanopathogenesis of lymphedema, how mechanical cues interact with biochemical cues to drive the onset and progression of lymphedema. Next, we examine the emerging AI-assisted diagnostic tools for lymphedema and its mechanical cues, which could offer earlier and more accurate detection compared to traditional methods. We then discuss AI-driven therapeutic interventions, including smart compression devices, reconstructive surgeries, and molecular mechanotherapeutic drugs. These innovative interventions use mechanical insights to improve patients’ prognosis. By unraveling the potential of mechanical cues in diagnostics and therapeutics (Table 1), we aim to inspire a perspective in which clinicians may increasingly leverage advances in AI and mechanobiology to address persistent clinical challenges of cancer-related lymphedema. Concomitantly, the ethical concern of AI-assisted diagnosis and therapy still needs to pay attention. Current AI-empowered lymphedema care must be designed as clinician-supervised decision support, not autonomous diagnosis or treatment allocation.

**Table 1.** Core mechanical biomarkers in cancer-related lymphedema

Biomarker	What it reflects	How it is measured	Approximate range	Main clinical use	Key limitation	References
Tissue stiffness	Fibrotic remodeling and loss of compliance	Ultrasound elastography, MRI elastography, skin meter	Roughly 3.15–12.80 kPa, but varies by tissue layer, anatomical site, stage, and device	Detecting fibrosis, staging, treatment selection	Cross-device variability and lack of unified cutoffs	[28–30,38]
Extracellular fluid (ECF) burden	Early fluid accumulation before over swelling	BIS	Direct interstitial/tissue fluid pressure is less standardized, around 2.5 ± 3.0 mmHg for obstructive lower-limb lymphedema; or BIS: L-Dex >10 or a ≥+6.5-unit increase from baseline	Surveillance and longitudinal monitoring	Influenced by hydration, BMI, and measurement context	[25,41,59]
Superficial lymphatic flow pattern	Residual lymphatic transport capacity and dermal backflow	ICG lymphography	Typical ICG progression: stage 0 = linear pattern/DB; stage I = splash; stages II–IV = stardust; stage V = diffuse	Early diagnosis and microsurgical planning	Operator dependence and limited standardization	[42,69]
Limb volume/shape asymmetry	Macroscopic disease burden	Perometry, 3D scanning, smartphone imaging	5–10% relative volume increase refers to early/subclinical warning; ≥10% relative limb-volume increase or ≥200 ml inter-limb volume difference as a diagnostic threshold in BCRL studies	Screening and follow-up	Less sensitive for preclinical disease	[8,39]

## Unraveling Mechanopathogenesis of Cancer-Related Lymphedema

**Fig. 1.**
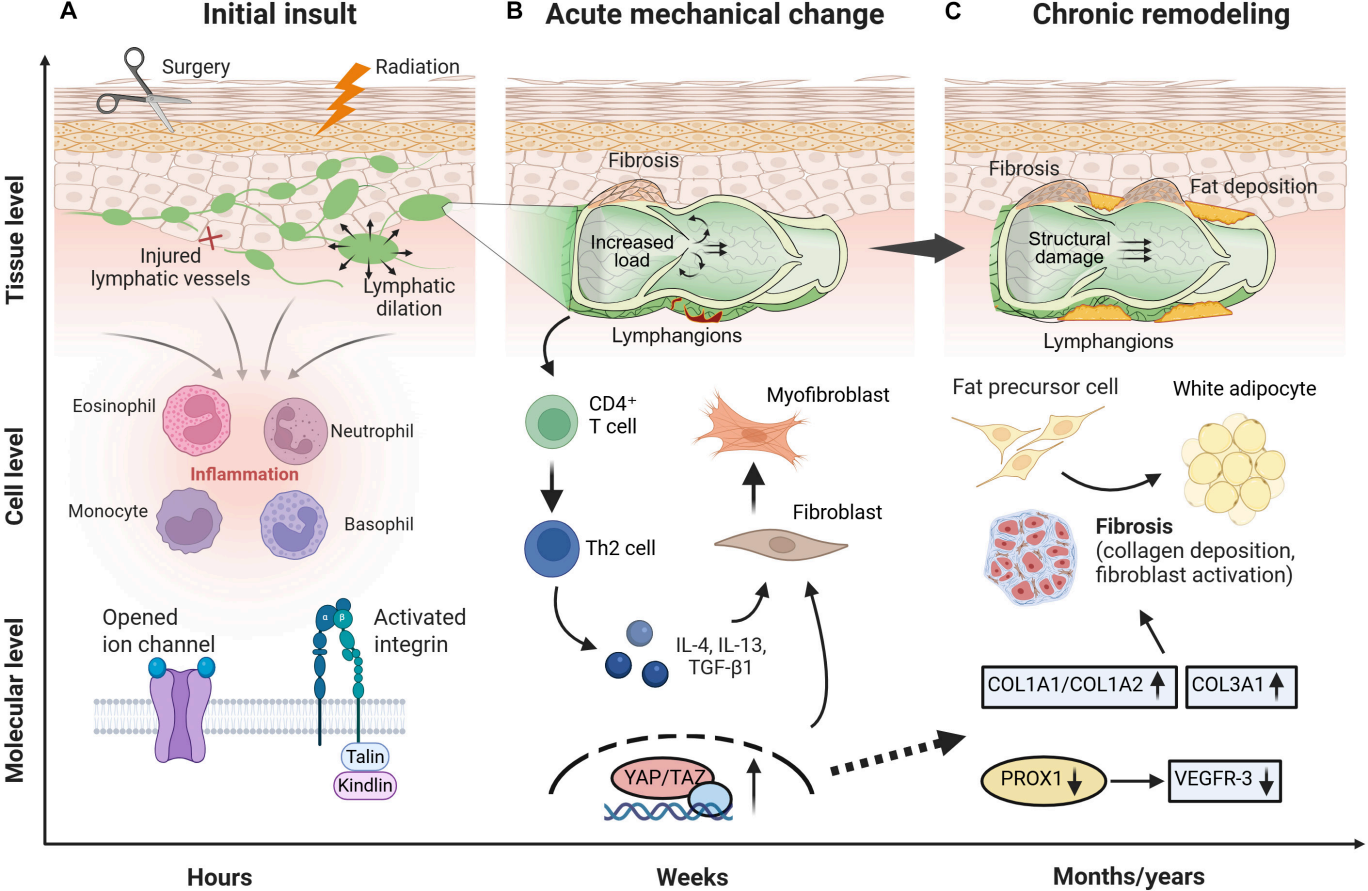
Cancer-related lymphedema driven by a self-reinforcing mechano-immune-fibrotic loop spanning molecular, cellular, and tissue scales. Cancer-related lymphedema evolves through a cascade of mechanically driven events spanning molecular, cellular, and tissue scales over distinct temporal phases. (A) Initial insult (hours). Cancer treatments (e.g., lymph node dissection or radiation) directly injure lymphatic vessels, leading to impaired lymph drainage, acute lymphatic dilation, and elevated interstitial fluid pressure. The resulting tissue stretch activates mechanosensitive pathways, including stretch-activated ion channels and integrin-based adhesions, while triggering early inflammatory responses characterized by innate immune cell infiltration. (B) Acute mechanical adaptation (weeks). Sustained lymphatic overload increases mechanical stress within lymphangions, initially inducing compensatory dilation and hypercontractility. Concurrently, protein-rich lymph stasis promotes a Th2-skewed immune milieu, with CD4^+^ T cells producing profibrotic cytokines such as IL-4, IL-13, and TGF-β1. These signals, together with increased matrix tension, activate fibroblasts and drive their differentiation into contractile myofibroblasts. Mechanotransduction pathways, particularly YAP/TAZ, integrate ECM stiffening and inflammatory cues to amplify profibrotic gene programs. (C) Chronic remodeling (months to years). Persistent mechanical overload and unresolved inflammation culminate in irreversible fibrofatty remodeling, characterized by excessive collagen deposition (up-regulation of COL1A1/COL1A2 and COL3A1), ECM crosslinking, adipose tissue expansion, and progressive structural damage of lymphangions. At the molecular level, sustained YAP/TAZ activity reinforces matrix production while suppressing lymphatic identity and regenerative capacity, as indicated by reduced PROX1 and VEGFR-3 signaling. Together, these multiscale processes establish a self-reinforcing mechanobiological loop that drives chronic tissue stiffening, lymphatic pump failure, and disease progression.

Cancer-related lymphedema should be understood as a dynamic mechanobiological disorder driven by sustained alterations in tissue mechanics, rather than just a passive fluid accumulation. Figure 1 summarizes how the disease progresses through different phases where mechanical forces interact with inflammatory and stromal responses, hindering lymphatic structure and function.

### Initial lymphatic injury and early mechanical perturbation

Cancer-related lymphedema typically initiates as an adverse effect of treatments like lymph node dissection or radiotherapy, which cause direct lymphatic injury [11,12]. These procedures disrupt lymphatic continuity, suddenly impairing lymph drainage and causing protein-rich interstitial fluid to build up. This buildup leads to increased interstitial fluid pressure, which infringes abnormal tensile and shear stresses on the remaining surrounding lymphatic vessels and tissues.

At the tissue level, this elevated pressure causes lymphatic tissue to enlarge over time and increases mechanical strain to nearby tissues. While at the cellular level, the endothelial junctions in damaged lymph vessels get mechanically strained and susceptible to leak [13,14]. At the same time, tissue deformation activates mechanosensitive pathways such as stretch-activated ion channels and integrin-based adhesions translate physical forces into intracellular signals [15,16]. These early mechanical cues trigger an acute inflammatory response caused by infiltration of innate immune cells, allowing for further pathological remodeling.

### Acute mechanical adaptation and profibrotic signaling amplification

As lymphatic dysfunction progresses, sustained lymphatic overload forces tissues into a phase of acute mechanical adaptation marked by altered pump mechanics. Lymphangions experience an increase in luminal pressure and wall tension, inducing compensatory dilatation and enhanced contractile activity. However, this adaptive response is metabolically demanding and mechanically unstable, which precipitates a functional decline.

Concurrently, accumulation of protein-rich lymph fluid causes chronic inflammation. CD4^+^ T cells within lymphedematous tissues preferentially adopt a Th2 phenotype [17,18], secreting profibrotic cytokines such as interleukin-4 (IL-4), IL-13, and transforming growth factor β1 (TGF-β1) [19]. These cytokines and the mechanical stress activate resident fibroblasts and promote their differentiation into myofibroblasts that express α-smooth muscle actin.

At the molecular level, mechanotransduction pathways become central integrators of these signals. The PECAM-1/VE-cadherin/vascular endothelial growth factor-3 (VEGFR-3) junctional complex in lymphatic endothelial cell membrane couples fluid shear stress to endothelial signaling and lymphatic remodeling [20], while PIEZO1-mediated calcium influx contributes to flow sensing, lymphatic valve development, and valve maintenance [21]. The transcriptional regulators FOXC2 and PROX1 further preserve lymphatic endothelial identity, collecting-vessel specialization, and valve integrity [22,23]. At last, together with YAP/TAZ and TGF-β signaling, a broad mechanosensing network is assembled, including junctional mechanoreceptors, stretch-activated ion channels, transcriptional programs, cytoskeletal tension, and extracellular matrix (ECM) feedback.

Within this network, the transcriptional co-activators YAP and TAZ are particularly sensitive to increased matrix stiffness and cytoskeletal contractility. Rather than functioning solely downstream of inflammatory signaling, YAP/TAZ operate in parallel with TGF-β pathways, amplifying profibrotic gene programs that include collagen synthesis, matrix crosslinking, and cytoskeletal reinforcement. TGF-β1, which is markedly elevated in lymphedematous tissues, is both profibrotic and anti-lymphangiogenic [24]. It drives fibroblast-to-myofibroblast differentiation, promotes excessive ECM production, and suppresses lymphatic regeneration by inhibiting lymphangiogenic signaling [25,26]. Consistent with this role, blockade of TGF-β1 in mouse models markedly reduces fibrotic scarring and improves lymphatic vessel regrowth [27]. Together, these findings highlight a convergence of mechanical and cytokine-driven signaling that establishes a feed-forward loop progressively biasing tissues toward fibrosis.

### Chronic fibrofatty remodeling and lymphatic failure

Lymphedema enters a new phase as prolonged mechanical overload and unresolved inflammation transitions into chronic irreversible remodeling of fibrofatty tissue. Clinically, longstanding lymphedema manifests as firm, fibrofatty swelling of the affected limb [28]. Hypoxia and sustained tissue tension can stimulate adipocyte differentiation and hypertrophy, a phenomenon also observed in other fibrotic disorders. In lymphedema, abnormal expansion of subcutaneous adipose tissue and a preadipocyte-to-adipocyte switch have been described in response to inflammatory and mechanochemically stressed microenvironments [29,30]. This adipose deposition further stiffens the tissue and may physically compress lymphatic vessels, compounding drainage impairment.

Excessive deposition of ECM proteins, particularly type I and type III collagens, leads to marked tissue stiffening and loss of compliance. Activated myofibroblasts persist within the interstitium, sustaining collagen production while limiting matrix degradation, thereby locking tissues into a high-stiffness state.

At the lymphatic endothelial level, sustained mechanical and inflammatory stress is associated with impaired lymphatic identity and regenerative capacity. Reduced expression of key lymphatic regulators, including PROX1 and VEGFR-3, correlates with diminished lymphangiogenic potential and progressive structural damage to lymphangions, including valve dysfunction and smooth muscle fatigue. Together, these changes culminate in lymphatic pump failure and persistent edema.

### A self-reinforcing mechanobiological loop

Across all stages, a unifying principle emerges: Mechanical forces and biological responses are tightly coupled in a self-reinforcing loop. Elevated interstitial pressure and matrix stiffening activate mechanosensitive pathways such as YAP/TAZ, which drive fibrosis and ECM accumulation. In fibrotic lymphedema, YAP/TAZ remain persistently active owing to increased matrix stiffness, thereby promoting continued matrix production and fibroblast activation [15,31]. Supporting this concept, a mouse tail model of lymphedema demonstrated that mesenchymal stem cells (MSCs) within lymphedematous tissues exhibit elevated YAP activity, and that modulation of YAP signaling alters disease outcomes [32]. Down-regulation of YAP in transplanted stem cells enhanced lymphatic growth through up-regulation of lymphatic-specific genes such as PROX1 and VEGFR-3 while simultaneously reducing fibrosis, resulting in functional improvement of lymphedema [18]. Consistent with these findings, early clinical studies suggest that local injection of bone marrow- or adipose-derived MSCs can reduce limb volume and improve lymphatic function in patients with secondary lymphedema [33,34].

Together, these observations implicate the YAP/TAZ mechanosensing pathway as a promising therapeutic target and exemplify the concept of mechanomedicine, in which modulation of cellular mechanoresponses can alter disease trajectories. In turn, matrix stiffening further amplifies mechanotransduction, perpetuating fibroblast activation, suppressing lymphatic regeneration, and locking tissues into a chronic pathological state.

In summary, the mechanical effects of lymphedema vary over time and scales. Some symptoms appear immediately after surgical intervention (e.g., lymphatic vasospasm or dilation), while others take months to years to develop (e.g., fibrosis and adipose deposition). Within weeks from the original lymphatic injury, overload starts compromising contractility, ultimately leading to smooth muscle fatigue and pump failure [11,35]. On the long run, remodeled lymphatic vessels start evidencing thickened wall and valve dysfunction due to the sustained high pressure and inflammation [36]. Altogether, mechanopathogenesis comprehends molecular alterations such as elevated levels of COL1A1 and ACTA2, cellular activation of fibroblasts and leukocytes, and tissue remodeling, including skin and subcutaneous tissue stiffening and altered limb biomechanics. We outlined the functions, properties, and usage of core 4 biomarkers during the development progress in Table 1. In addition, we also tried to summarize their approximate quantification ranges, although the field lacks standardized thresholds across devices or cohorts. These biological alterations are not intended to serve as direct AI inputs in routine clinical care, but as mechanistic labels, covariates, endpoints, or validation targets for AI model development. In this framework, AI links measurable clinical signals, such as imaging, bioimpedance, and wearable-derived features, with underlying mechanical and biological lymphedema phenotypes.

It is worth noting that patient-specific factors (e.g., genetic predispositions or comorbidities like obesity) can modulate these mechanobiological pathways. Obesity, for example, increases interstitial fluid production and inflammation, which may explain why obese cancer survivors have a higher incidence and severity of lymphedema [37]. Likewise, radiation therapy can cause direct fibrosis and impair tissue compliance, accelerating mechanical failure of lymphatic drainage [38]. These factors aside, the unifying theme is that mechanical forces and biological responses are locked in a reinforcing cycle in lymphedema. Breaking this cycle, by reducing pathologic mechanical stress or blocking its fibrotic consequences, is key to improving outcomes.

It is important to distinguish between 2 complementary roles of AI in lymphedema research. The majority of current AI applications fall into the category of pattern recognition, where models classify, segment, or detect features in imaging or clinical data without explicitly modeling underlying biological mechanisms. Examples include convolutional neural network (CNN)-based classification of ICG lymphography patterns, radiomics-based severity grading, and risk prediction from clinical variables. While these approaches provide clinical utility, they generate statistical associations rather than mechanistic understanding.

A second, less mature but conceptually important role involves AI-supported mechanistic insight generation. This includes integrating multi-scale data to identify mechanobiological signatures, linking imaging features to specific tissue or molecular phenotypes, or using AI to discover patterns that suggest new biological hypotheses. For instance, AI models that connect tissue stiffness measurements with downstream YAP/TAZ activity or fibroblast activation states would represent mechanistic integration rather than pattern recognition alone. At present, most reported applications in lymphedema fall into the pattern recognition category, and the development of AI tools that generate genuine mechanistic insight remains an open challenge for the field.

## AI-Enhanced Mechanical Signatures Diagnostics and Intelligent Analysis

**Fig. 2.**
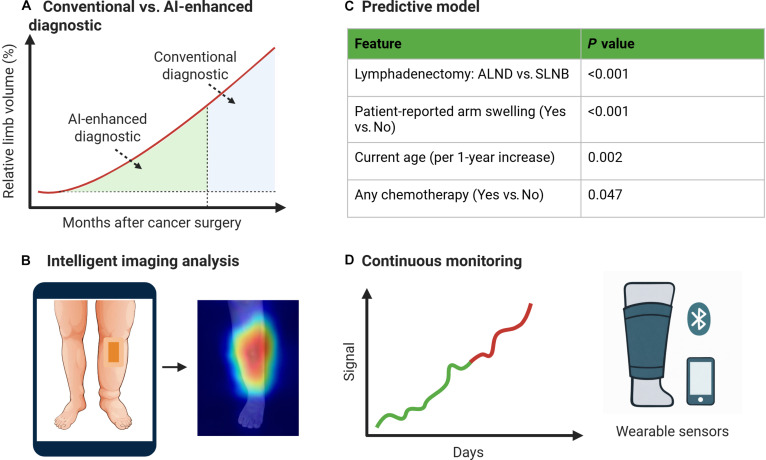
AI-enhanced diagnostics for early detection and monitoring of cancer-related lymphedema based on mechanical signatures. (A) Conventional versus AI-enhanced diagnosis. The green shaded region illustrates the diagnostic time window gained through AI-assisted analysis before clinically evident volume increase. (B) Intelligent imaging analysis. AI-based pattern recognition enables objective identification of early lymphatic dysfunction and tissue stiffening that may not be readily discernible by visual inspection alone. (C) Data-driven predictive models [81]. Here is a validated machine learning-based clinical prediction model reported in *JAMA Network Open*. (D) Continuous monitoring and longitudinal assessment. Wearable sensors and mobile health platforms generate continuous, time-resolved data on limb mechanics and fluid status for AI-based analysis.

Cancer-related lymphedema often develops insidiously, and early detection has been a persistent clinical challenge. Traditionally, diagnosis relies on gross limb measurements (circumference or volume changes) or qualitative signs (e.g., the Stemmer’s skin fold sign) after swelling becomes evident. However, by the time visible swelling is present, significant mechanical damage, fibrosis and lymphatic dysfunction, may already have occurred [8]. In recent years, advanced imaging techniques and AI-powered algorithms have begun to unveil subtle mechanical signatures of lymphedema before it reaches overt stages (Fig. 2A). Across available modalities, AI is not a single tool but a set of task-specific analytical strategies that translate mechanical or functional signatures into clinically actionable outputs (Table 2). Here, we discuss how AI is augmenting diagnostic modalities, from medical imaging to risk prediction models, enabling earlier and more accurate identification of lymphedema in cancer survivors.

**Table 2.** AI-enabled diagnostic and predictive tasks for cancer-related lymphedema

Input modality	Typical AI task	Clinical output	Main strengths	Main limitations	References
Ultrasound elastography	Classification, staging, longitudinal change detection	Early fibrosis detection; fluid-dominant vs. fibrosis-dominant stratification	Noninvasive; easily available; directly aligned with mechanobiology	Device dependence; variable acquisition protocols; limited standard thresholds	[40,41]
MRI/radiomics	Severity prediction, classification, progression modeling	Severity grading; fibrosis burden estimation	High anatomical detail; deep tissue features	Expensive; limited accessibility; relatively small datasets	[47,48]
ICG lymphography	Pattern classification, segmentation, severity scoring, surgical mapping	Early abnormal flow detection; microsurgical planning	Highly informative for lymphatic function; useful in early disease	Requires dye injection; operator experience needed^,^	[42,67,69]
Smartphone imaging	Image classification, segmentation, remote screening	Scalable screening tool; home-based monitoring	Easily accessible; supports telemedicine	Sensitive to lighting, angle, skin tone, and image quality	[51]
Perometry/3D scanning	Automated quantification, progression tracking	Objective swelling trajectories	Established quantitative endpoint; easier standardization than photos	Detects relatively late macroscopic change	[39,41,48]
BIS	Alerting, longitudinal trend prediction, risk modeling	Preclinical edema surveillance; flare detection	Sensitive for surveillance; practical repeated measurement	Nonspecific; influenced by physiology and measurement context	[41,59]
Wearables	Time-series forecasting, anomaly detection, digital phenotyping	Early warning of exacerbation or non-adherence	Enables continuous monitoring and personalized feedback	Data sparsity, adherence burden, consumer-device variability	[10,60,61]
Multimodal ML models	Risk prediction, response prediction, prognosis	Individualized lymphedema risk score	Most clinically realistic; can combine baseline and dynamic data	Data harmonization, missingness, external validation challenges	[47,48,50–53,81]

### Imaging the invisible

One fruitful approach uses imaging techniques that capture tissue biomechanics or lymphatic function, paired with AI for pattern recognition. For example, tissue elastography [ultrasound or magnetic resonance imaging (MRI)-based] can quantify skin and subcutaneous tissue stiffness [39]. Lymphedematous limbs show higher shear modulus (i.e., stiffer tissue) compared to normal limbs due to fibrotic changes. In a study using ultrasound elastography, a threshold stiffness could distinguish early-stage breast cancer-related lymphedema from a healthy arm, aiding staging of the disease [40]. AI algorithms have been applied to such elastography data to automatically classify lymphedema severity with promising accuracy [41]. Similarly, indocyanine green (ICG) lymphography is an imaging technique where a fluorescent dye maps superficial lymphatic flow. Clinicians recognize characteristic dermal backflow patterns on ICG images that signify lymphatic obstruction (e.g., splash, stardust, and diffuse patterns). Recently, machine learning models have been trained to interpret ICG lymphography images, effectively teaching a computer to identify these complex patterns. One recent study demonstrated that an AI model could classify ICG lymphographic patterns with high accuracy, standardizing what was once a subjective interpretation [42]. By automating ICG image analysis, AI can flag abnormal lymphatic flow even when subtle, providing an objective diagnostic aid. Beyond static image interpretation, dynamic lymphatic imaging provides an important opportunity for AI-enabled functional assessment [42,43]. Near-infrared fluorescence ICG lymphography can capture lymphatic propulsion, dermal backflow expansion, tracer migration, and collecting-vessel pumping activity over time [44]. These lymphodynamic data may be converted into quantitative features, such as contraction frequency, contraction amplitude, transport velocity, reflux pattern, and clearance kinetics. AI models trained on such time-resolved functional parameters could help distinguish fluid-dominant early dysfunction from advanced pump failure, predict progression risk, and guide individualized selection of compression, rehabilitation, or lymphatic reconstruction strategies [45,46].

AI is also being leveraged in cross-sectional imaging and computer vision. MRI and computed tomography (CT) scans of lymphedema patients sometimes show increased subcutaneous fat and thickened skin. Radiologists have explored radiomic analysis, extracting quantitative features from MRI (like texture and gradient patterns in tissues), combined with machine learning to detect lymphedema and even predict its stage [47,48]. Meanwhile, simple clinical photographs of a patient’s limbs can harbor clues: differences in limb contour, skin folds, or redness. Cutting-edge deep learning algorithms (convolutional neural networks) have been trained on clinical images to differentiate lymphedema from other causes of leg edema (such as venous edema or heart failure) (Fig. 2B). In a recent study with over 1,600 leg images, a deep learning model reached ~79% accuracy in classifying lymphedema versus other edema conditions, outperforming human clinicians (~63% accurate) [8]. Notably, visualization techniques (like Grad-CAM heatmaps) showed AI focused on regions of subtle skin thickening and hyperpigmentation, features of chronic lymphedema, demonstrating that the model identified mechanical or morphological changes that experts sometimes overlook. This kind of tool could be deployed via a smartphone app for community screening, where a survivor’s limb photo is analyzed instantly for early lymphedema signs. Beyond detecting lymphedema, AI-based segmentation can estimate tissue composition. Automated CT segmentation can quantify subcutaneous fluid, adipose expansion, muscle compartments, skin thickening, and fibrosis-associated tissue changes, thereby supporting a transition from binary diagnosis to compositional mechanophenotyping [49]. This is particularly relevant because fluid-dominant, fat-dominant, and fibrosis-dominant lymphedema may require different therapeutic strategies.

### Data-driven risk prediction

Beyond imaging, AI begins to show the potential to transform risk assessment by integrating diverse patient data. Large cohorts of breast cancer survivors have been used to train machine learning models that predict an individual’s likelihood of developing lymphedema (Fig. 2C) [50–53]. These models incorporate mechanical risk factors (extent of surgery, radiation-induced tissue scarring) alongside clinical variables [body mass index (BMI), infections, etc.). For example, an extreme gradient boosting (XGBoost) model was recently developed using data from 240 breast cancer patients, which achieved an area under the receiver operating characteristic (ROC) curve (AUC) of 0.89 in validation, identifying patients at high risk for lymphedema after surgery [52]. Significant predictors included the number of lymph nodes removed (which correlates with lymphatic mechanical load) and whether patients performed postoperative arm exercises (affecting lymph flow). However, these findings should be interpreted cautiously. The study was based on a relatively small, single-center retrospective cohort and lacked external validation. Moreover, the decline in model performance from the training to the validation set suggests possible overfitting. Future studies should validate such models in larger, multicenter prospective cohorts and explicitly address potential sources of selection, measurement, and confounding bias. Another study took an innovative approach by using nontraditional data: They fed a machine learning model with patients’ routine blood test results and treatment details, without relying on limb measurements or symptoms [54]. Impressively, with data from ~2,100 patients, their random forest model reached a balanced accuracy of ~87% in detecting lymphedema, and an AUC of 0.93. This suggests that there are subtle biological signals (perhaps inflammatory or metabolic markers in blood) associated with the mechanical pathology of lymphedema that AI can discern. The researchers even created a web-based tool for clinicians to input a patient’s lab values and get an instant lymphedema risk assessment. Such tools exemplify how AI can synthesize multiple weak indicators into a strong predictive signal.

### Personalized monitoring

For patients already diagnosed with early lymphedema, AI can assist in monitoring and thereby improve outcomes. Wearable technologies (e.g., smart compression sleeves and bioimpedance sensors) generate continuous data on limb fluid and circumference changes. AI algorithms can analyze these time-series data to detect exacerbations or suboptimal control (Fig. 2D) [55]. Additionally, patient-reported symptoms collected via smartphone apps can be analyzed with machine learning to correlate subjective reports with objective swelling. A pilot study used a machine learning classifier on real-time symptom diaries of breast cancer survivors and was able to detect lymphedema flares with >90% sensitivity [56]. By alerting clinicians to a patient’s deteriorating status before the next clinic visit, such systems enable timely interventions (e.g., adjusting compression therapy or adding physical therapy).

It is important to note that AI-aided diagnosis is meant to complement, not replace, clinical evaluation. The best results often come from hybrid models: For example, a recent study developed 2 predictive models for breast cancer-related lymphedema, one preoperative and the other postoperative, combining easy-to-measure clinical factors (e.g., age, BMI, and extent of surgery) with known racial disparities in incidence [57]. These logistic models (which are simpler than black-box AI) achieved good accuracy (AUC ~0.78 to 0.86) and were externally validated for clinically usable, pending implementation-impact testing. They underscore that known risk factors (e.g., axillary lymph node dissection, which multiplies risk ~3.6-fold) still play a central role. The promise of AI is to integrate such known factors with new data (e.g., imaging, genomics, and real-time monitoring) to refine predictions.

Across these studies, AI is best understood not as a single diagnostic tool but as a set of task-specific analytical strategies that translate mechanical or functional signatures into clinically actionable outputs. The main input modalities, AI tasks, clinical outputs, and current translational gaps are summarized in Table 2. Ultimately, early identification through AI-enhanced diagnostics opens the door for early intervention, and early intervention is repeatedly shown to limit lymphedema severity. By catching the mechanical dysfunction in its infancy (e.g., slight increases in tissue fluid or stiffness), clinicians can initiate therapies that prevent progression to irreversible fibrosis.

## Mechanotherapy and AI-Guided Interventions

Management of cancer-related lymphedema has historically focused on symptomatic relief, for example, using compression bandage, manual lymphatic drainage massage, and exercise to reduce swelling. While these conservative therapies improve quality of life, they do not cure the underlying lymphatic dysfunction or reverse tissue fibrosis [58]. In recent years, however, a wave of mechanobiology-informed interventions has begun to target the root causes, trying to rebuild or reroute lymphatic flow, soften fibrotic tissue, and re-educate the affected limb’s biology. Here, we discuss how mechanomedicine concepts are guiding the development of novel approaches for lymphedema and how AI is being explored to assist in personalizing and optimizing these therapies, noting that most applications remain far from being ready for deployment.

### Smart compression and mechanostimulation

**Fig. 3.**
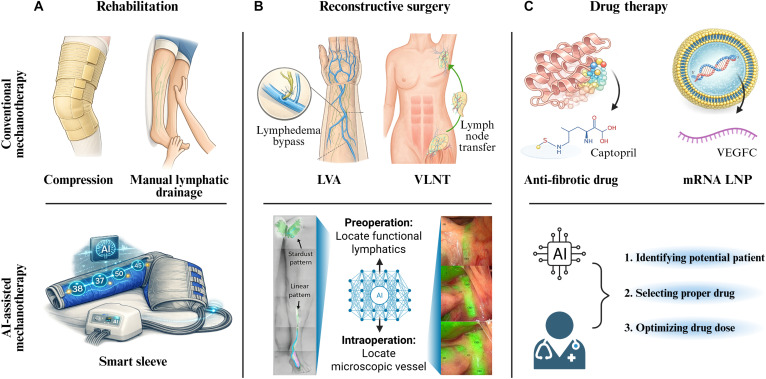
Mechanophenotype-informed management strategies and potential AI support in cancer-related lymphedema. (A) For rehabilitation, conventional mechanotherapies, e.g., compression and manual lymphatic drainage, have been updated with AI-guided smart sleeve. (B) For reconstruction surgery, conventional mechanotherapies, e.g., LVA and VLNT, have been improved by AI-assisted lymphatic imaging and functional assessment from preoperations or intraoperations. (C) For drug therapy, emerging mechanotherapy drugs, e.g., anti-fibrotic molecules and growth factor mRNA, and potential AI-assisted personal precise therapy arise.

Compression therapy remains the cornerstone of lymphedema care, where it applies external pressure to counteract internal fluid pressure and mechanically propel lymph. Early-stage AI integrations are being explored to make compression smarter and more adaptive. For instance, recent pilot implementations of new AI-optimized pneumatic compression devices may achieve volume reductions comparable to conventional compression drainage. However, these findings come from small, sometimes single-center studies and require larger prospective validation (Fig. 3A). Sensors planted in the sleeve can measure limb volume or stiffness in real time, and an AI algorithm dynamically adjusts pressure in turn to ensure optimal lymphatic pumping without causing discomfort [59]. These “smart sleeves” can target specific areas (e.g., tighter pressure where edema is greatest) and even adapt with the patient’s breathing or muscle contractions to enhance fluid return, which is ready for home-based or clinically used [60]. Early pilot studies suggest that AI-optimized compression plan can achieve volume reductions comparable to expert manual therapy, but with greater convenience (e.g., at-home use) and consistency [61]. Another emerging therapy manner is vibrational or oscillatory stimulation of tissues, as an advanced version of massage therapy. By applying high-frequency low-amplitude vibrations to the limb, this manner can enhance interstitial fluid movement and fibroblast activity. Some physical therapy clinics now use vibratory plates or handheld devices to soften fibrotic tissue, wherein the mechanical stimulus is thought to disrupt stiff collagen crosslinks [62,63]. While still experimental, there are reports that adjunct vibration therapy improved skin elasticity and reduced tightness in patients with chronic lymphedema. Similarly, AI could play a role here by optimizing vibration frequency and duration per patient, essentially tuning the mechanical signal to the tissue’s response. Besides compression therapy, some physical therapy methods were recently added as supplement of conventional compression therapy to improve lymphatic reflux and limb swelling, e.g., medium-frequency electrotherapy, airwave pressure therapy, and ultrasonic therapy [64].

### Reconstructive surgery and AI planning

For moderate to severe lymphedema, surgical interventions are increasingly utilized. Two main surgical approaches are lymphatic venous anastomoses (LVA), supermicrosurgery connecting lymphatic vessels to veins to bypass obstructed lymph pathways, and vascularized lymph node transfer (VLNT), transplanting healthy lymph nodes, often from the abdomen, into the affected limb to rebuild lymphatic drainage (Fig. 3B) [65,66]. The success of these delicate procedures depends on selecting the right patients and precisely locating functional lymphatic channels or suitable recipient vessels. Here, AI is being explored to assists surgeons during preoperative mapping. ICG lymphography images, as mentioned, have been explored for AI-assisted localization of functional lymphatics [42,67]. Similarly, ultrahigh-frequency ultrasound combined with AI image segmentation may help differentiate patent from scarred vessels, although these applications persist at early development stages. Some teams are based on MRI or ICG data developing augmented reality software that overlays mapped lymphatics onto the patient during surgery, reducing guesswork in locating microscopic vessels. Additionally, predictive analytics guide surgical decision-making [68,69]. For example, an AI model might predict which patients are likely to benefit most from VLNT or LVA, based on features like distribution of fibrosis or presence of distal versus proximal obstruction [70]. The above studies demonstrate the potential of AI to support reconstructive surgical planning. However, further clinically oriented applications and well-designed multicenter trials are needed to validate these tools, establish their generalizability, and facilitate their translation into routine clinical practice.

In addition, robot-assisted lymphatic reconstructive surgery is emerging as enabling technology for complex supermicrosurgical procedures. Robotic lymphovenous anastomosis (RoboLVA) has demonstrated technical feasibility in patients with lymphedema, with short-term outcomes comparable to those of conventional microsurgery [71]. First-in-human case reports have further described how such systems may improve precision and accessibility in lymphatic microsurgery [72]. Notably, the potential integration of AI into RoboLVA represents a promising research direction, which warrants systematic investigation in both translational research and clinical settings.

### AI-assisted drug mechanotherapy

Targeting the fibrotic microenvironment, mechanomedicine has opened the possibility of pharmacological interventions that alter the tissue mechanics. One promising area is anti-fibrotic or anti-inflammatory drugs repurposed for lymphedema (Fig. 3C). For example, TGF-β1 inhibitors are being explored to prevent or reduce lymphedema fibrosis. Angiotensin-converting enzyme (ACE) inhibitors like captopril, which have anti-TGF-β effects, showed in a mouse lymphedema model that topical captopril reduced skin fibrosis and improved lymphatic function [73]. A small human pilot trial hinted that oral ketoprofen, an anti-inflammatory that also inhibits fibrosis pathways, led to softer tissue and improved limb volume in lymphedema patients over 4 to 6 months [74]. While not yet standard of care, these examples illustrate an emerging shift toward treating the mechanical microenvironment of lymphedema in addition to just fluid management. Unlike imaging-based diagnosis or risk prediction, the application of AI to pharmacological mechanotherapy remains at a very early stage. Several studies reported that AI can analyze patient datasets to verify if patients on certain medications (like anti-fibrotics or anti-inflammatory) had slower lymphedema progression [59,75]. Such real-world evidence identified through AI pattern analysis can promote drug repurposing trials.

Targeting the regeneration of lymphatic vessels, growth factor therapy is at the forefront here. A 2021 preclinical study demonstrated that delivering VEGF-C, a key lymphangiogenic factor, via an mRNA-loaded nanoparticle induced robust growth of new lymphatic vessels in mouse models and reversed lymphedema phenotypes (Fig. 3C) [76]. A single low dose of VEGF-C mRNA was enough to form functional lymphatic networks and restore fluid drainage in the lymphedematous limb, with effects lasting months. Importantly, this preclinical therapy improved limb swelling and reduced fibrosis without significant adverse effects, illustrating an approach aligned with mechanomedicine. There is already a phase I clinical trial of a VEGF-C gene therapy (Lymfactin) in combination with lymph node transfer, which showed safety and some encouraging signs of efficacy in patients [77]. Notably, a recent translational study reported that anti-CTLA4 therapy was associated with a markedly reduced risk of lymphedema in melanoma patients after lymphadenectomy and experimentally reduced edema while improving lymphatic function in mouse tail lymphedema models, potentially through systemic and local expansion of FOXP3^+^ regulatory T cells [78]. This study provides a human-first translational example linking clinical observation, immune mechanism, and animal validation. Looking ahead, AI may eventually support the evaluation of regenerative therapies by integrating imaging-based outcomes, clinical trajectories, and trial-level data.

Beyond VEGF-C and CTLA4, other molecular targets are emerging from mechanobiology research. For instance, targeting YAP/TAZ or downstream pathways could potentially tilt the balance from fibrosis toward regeneration [32]. Similarly, modulating Piezo1 channels, mechanosensitive ion channels on lymphatic endothelium, might enhance lymphatic pumping. There is evidence that Piezo1 helps lymphatics sense fluid shear stress [15]. These ideas are still at an early stage, but they illustrate how understanding the mechanical underpinnings yields new therapeutic avenues.

### AI for personalizing therapy

The main challenge of lymphedema therapy is its heterogeneous nature. In some patients, inflammation dominates; in others, fibrosis and some may present a more proximal or distal fluid stasis. This heterogeneity argues for a mechanophenotype-guided treatment framework, in which AI supports future treatment matching, planning, and monitoring (Table 3). In principle, AI could potentially analyze a patient’s imaging and molecular data to support clinical decisions, for example, by suggesting that patient A, characterized by high tissue stiffness and active TGF-β signaling, might benefit from an anti-fibrotic drug or mechanical microenvironment-driven reprogrammed cells [79]. Patient B, with minimal fibrosis but significant fluid, might benefit more from aggressive physical therapy and perhaps early LVA surgery. Such AI approaches are conceptually appealing, although they remain largely theoretical and require substantial clinical validation before integration into routine care. Early versions of this approach are seen in decision support systems under development. One prototype tool integrates patient risk factors and preferences to recommend either conservative management or referral for surgical consultation and was shown to improve guideline adherence in a simulated study.

**Table 3.** Mechanophenotype-guided interventions and the role of AI in precision mechanomedicine

Patient phenotype	Representative features	Candidate intervention	Role of AI	Expected benefit	Evidence maturity	Major translational barrier	References
Preclinical/ high-risk	Elevated ECF, subtle stiffness change, abnormal early flow	Early compression, exercise, remote monitoring	Risk prediction	Earlier intervention	Moderate	Need prospective validation and standardized thresholds	[53,54,81]
Fluid-dominant	Reversible swelling, limited fibrosis	Compression therapy, smart sleeves, manual lymphatic drainage, exercise	Adaptive pressure control, adherence monitoring, symptom forecasting	Progression control and even improved	Moderate	Limited integration of wearables into routine care	[59–62,74]
Fibrosis-dominant	High stiffness, thickened tissue, reduced compliance, late-stage remodeling	Anti-fibrotic therapy, rehabilitation, staged combined treatment	Fibrosis quantification, treatment response prediction, phenotype classification	Rational selection of anti-fibrotic or regenerative approaches	Low to emerging	Few validated fibrosis biomarkers and limited interventional data	[73]
Mixed fluid–fibrotic phenotype	Combined edema and stiffness abnormalities; heterogeneous local mechanics	Combination therapy (compression + rehabilitation + selective surgery or drug therapy)	Multimodal phenotyping, decision support, adaptive treatment sequencing	Personalized therapy	Emerging	Requires multimodal data integration and clinically interpretable models	[63,64]
Surgery-amenable	Residual functional collectors on ICG, localized obstruction, potentially reversible transport	Lymphovenous anastomosis (LVA)	Surgical planning, vessel/target identification, candidate selection	Selection of proper microsurgical intervention	Moderate	Need standardized imaging-AI workflows and external validation	[65,69,70]
Advanced refractory disease	Extensive dermal backflow, poor collectors, marked fibrosis, severe chronicity	VLNT, debulking/liposuction, multimodal long-term care	Candidate selection, preoperative mapping, outcome prediction	More appropriate referral and realistic expectation setting	Moderate	Limited multicenter prospective evidence	[65,66]
Regeneration-amenable phenotype	Incomplete failure, residual regenerative potential, modifiable microenvironment	VEGF-C-based strategies, cell therapy, lymphangiogenic/regenerative approaches	Patient stratification, response prediction, biomarker-guided enrichment	Enables mechanism-based intervention	Low/experimental	Need biomarker-defined responder groups and translational safety data	[56,57,76,77]
Long-term self-management phenotype	Chronic fluctuating course, adherence-dependent outcomes, recurrent swelling episodes	Home monitoring, AI coaching, digital rehabilitation, self-care optimization	Personalized reminders, behavior analytics, adherence support	Improves sustainability of care and patient engagement	Emerging	Engagement fatigue, reimbursement, workflow integration	[68,80]

Finally, it is worth touching on patient engagement and behavioral interventions. Lymphedema often requires lifelong self-care, including daily sleeve use, skin care, and exercise. AI-driven smartphone apps can coach patients, using computer vision to check if a compression wrap is applied correctly, or using chatbots to encourage adherence. A recent pilot study of a “lymphedema virtual coach” AI reported better adherence to daily exercises and self-drainage massage among app users, with preliminary evidence of improved limb outcomes over 6 months [80]. These findings, while encouraging, derive from a single small pilot and require larger prospective validation. Thus, AI may complement high-tech interventions as well as low-tech measures through education and motivation.

In summary, mechanomedicine concepts are gradually expanding the landscape of cancer-related lymphedema therapeutics from a purely symptomatic management toward approaches that may eventually target the underlying disease mechanisms. Although mechanomedicine principles have begun to inspire strategies aimed at improving lymphatic regeneration, reducing fibrosis, and restoring tissue mechanics, most such interventions remain at preclinical or early clinical stages. The combination of AI-guided analysis and mechanobiology-based treatments may, with proper validation, contribute to improved long-term management of lymphedema.

## Conclusion and Future Perspectives

Cancer-related lymphedema, particularly in cancer survivors, exemplifies a complex condition at the intersection of mechanics and biology. Our review highlights that mechanical cues, such as tissue pressure, stiffness, and fluid flow, are key drivers of lymphedema’s onset and progression. Over the last few years, recognizing these mechanical hallmarks has led to initial progress in early identification of biomechanical abnormalities through AI-enhanced diagnostics and interventions targeting the root causes of the disease, although most approaches remain in early translational stages.

However, the comprehensive and widespread applications of AI in the diagnosis and treatment of lymphedema remain at an early translational stage. A major limitation is dataset heterogeneity, where available datasets differ substantially in patient population, cancer type, surgical procedure, disease stage, imaging modality, acquisition protocol, annotation standard, and outcome definition. For example, ICG lymphography datasets are often small, single-center, and manually annotated, which limits model generalizability. Domain shift is another critical challenge, because AI models trained on data from one institution, imaging device, ethnic group, or clinical workflow may perform poorly when applied to external cohorts. Therefore, multicenter external validation and prospective clinical testing are essential before AI-assisted diagnostic or therapeutic tools can be considered clinically reliable. In addition, many current AI models remain insufficiently explainable. Black-box predictions may be difficult for clinicians to interpret, especially when models are used for risk stratification, surgical planning, or treatment selection. Explainable AI methods, uncertainty estimation, and clinically interpretable feature attribution should therefore be incorporated to ensure that model outputs are biologically plausible and clinically actionable.

Regulatory barriers also need to be addressed, including data privacy protection, algorithmic bias, model updating, quality control, liability, and approval pathways for AI-based medical devices. Moreover, AI-assisted lymphatic surgery, adaptive compression systems, digital twins, and AI-guided pharmacological interventions still lack extensive prospective validation. Thus, although AI offers substantial potential for lymphedema mechanomedicine, its clinical implementation should proceed cautiously through standardized data collection, transparent reporting, external validation, regulatory evaluation, and ethically responsible deployment.

To clearly recognize the current advances of AI-empowered mechanomedicine, we summarize and classify the introduced clinical studies in this paper into 3 types, i.e., preclinical proof-of-concept, early clinical feasibility, and validated clinical approach (Table 4). As Table 4 illustrates, most current studies remain at preclinical proof-of-concept or early clinical stages, with relatively few validated clinical approaches. Acknowledging these limitations, the integration of AI and mechanobiology offers conceptual frameworks that hold great promise for enabling clinicians and researchers to tackle previously intractable questions in the future. Especially in the AI-empowered precise therapy of lymphedema, several exciting directions are outlined here with the understanding that most require substantial further validation before clinical translation.

• AI-empowered precise diagnosis. The first direction is the development of integrated precision diagnostic frameworks based on multimodal patient data. Instead of relying on single indicators such as limb circumference or isolated imaging findings, emerging AI approaches may integrate clinical history (e.g., extent of lymph node dissection and radiotherapy exposure), patient-reported symptoms and functional scores, imaging-derived features from modalities such as ultrasound elastography and lymphatic imaging, as well as laboratory-based inflammatory markers when available. By combining these heterogeneous inputs, machine learning models may enable more refined risk stratification and earlier identification of subclinical lymphatic dysfunction, potentially distinguishing fluid-dominant and fibrosis-dominant phenotypes. Importantly, these systems should be regarded as clinical decision-support tools requiring rigorous external validation, rather than autonomous diagnostic systems.
• Mechanotransduction pharmacology. The next few years will likely see clinical trials targeting key mechanosensitive regulators such as TGF-β and the YAP/TAZ axis. If successful, these efforts could contribute to lymphedema management by reducing tissue stiffening, although halting fibrotic progression at early stages remains an unproven clinical goal. In the future, AI-driven screening may help accelerate the discovery of these treatments by identifying drugs that specifically reverse the signature gene expression of lymphedema fibroblasts, while computational image analysis could support more objective and accelerated evaluation of efficacy.
• AI-driven rehabilitation. Beyond early diagnostic and treatments, AI may contribute to long-term lymphedema care. AI powered intelligent wearables are being explored as personalized physical therapy agents, autonomously adjusting mechanical and electrical stimuli based on real-time biometric analysis. These AI-driven personalization may help improve patient adherence to exercise regimens designed to facilitate lymphatic drainage. However, robust clinical evidence demonstrating that such behavioral interventions improve clinical outcomes remains limited and they represent a key area for prospective validation.

**Table 4.** Classification summary of clinical studies about AI-empowered mechanomedicine

Clinical study	Evidence type	Classification	Reference
Liposuction for swelling in lymphedema	Clinical therapeutic report/commentary	Validated clinical approach	[1]
Rehabilitation interventions for head and neck cancer-associated lymphedema	Systematic review of clinical rehabilitation interventions	Validated clinical management domain	[2]
Deep learning classification of lymphedema and other lower-limb edema diseases using clinical images	Clinical image-based AI diagnostic study, >1,600 images	Early clinical feasibility	[8]
Autologous bone marrow stromal cells for breast-cancer-related arm lymphedema	Prospective controlled clinical study	Early clinical feasibility	[33]
Autologous stem cells for post-mastectomy lymphedema	Pilot clinical study	Early clinical feasibility	[34]
Breast lymphedema after adjuvant radiation therapy	Clinical risk-factor study	Preclinical proof-of-concept	[38]
Elastography in diagnosis and staging of BCRL	Clinical diagnostic study	Early clinical feasibility	[40]
AI-based ICG lymphography pattern classification	Clinical AI image-classification study	Early clinical feasibility	[42]
Dynamic/static ICG lymphography for lymphatic dysfunction	Clinical imaging/functional assessment study	Early clinical feasibility	[43]
Noncontrast MRI-based ML/radiomics for lower-limb lymphedema severity	Clinical radiomics/ML study	Early clinical feasibility	[47]
Automated CT segmentation for lower-extremity tissue composition	Deep-learning segmentation study	Early clinical feasibility	[49]
ML models predicting BCRL risk in Chinese survivors	Cross-sectional ML model study	Early clinical feasibility	[50]
ML detection of lymphedema among breast cancer survivors	mHealth/symptom-based ML detection study	Early clinical feasibility	[51]
ML predictive modeling of BCRL; XGBoost, AUC 0.89 validation	Single-center retrospective ML model	Early clinical feasibility	[52]
Personalized ML risk prediction for BCRL after surgery	Recent ML risk model	Early clinical feasibility	[53]
Predictive models using blood tests and therapy data	Clinical ML model with routine laboratory/treatment data	Early clinical feasibility	[54]
AI prediction after immediate lymphatic reconstruction using synthetic data	Synthetic-data AI approach	Preclinical/computational proof-of-concept	[56]
Prediction models for BCRL incorporating racial differences	Clinical prediction model with validation	Validated clinical prediction model	[57]
External validation of a 5-factor BCRL risk model	External validation study	Validated clinical prediction model	[81]
Advanced pneumatic compression devices	Clinical outcome/health-economic study	Validated clinical device class	[60]
MOBIDERM Autofit nighttime compression sleeve	Pilot randomized controlled trial	Early clinical feasibility	[61]
Vibration added to simple lymphatic drainage	Randomized crossover pilot study	Early clinical feasibility	[62]
Compression-vibration combination device	Exploratory device study	Early clinical feasibility/exploratory	[63]
Complex physical therapy and multimodal approaches	Systematic review/meta-analysis of RCTs	Early clinical feasibility	[64]
Lymphatic ultrasound and multipoint ICG lymphography for LVA	Clinical surgical-planning workflow	Early clinical feasibility	[69]
Lymphoscintigraphic findings predicting LVA outcome	Retrospective cohort study	Early clinical feasibility	[70]
Robotic-assisted lymphovenous anastomosis	Systematic review of surgical outcomes	Early clinical feasibility	[71]
First-in-human robotic supermicrosurgery for BCRL	Randomized pilot trial	Early clinical feasibility	[72]
Topical captopril for secondary lymphedema	Preclinical/translational drug study	Preclinical proof-of-concept	[73]
Ketoprofen anti-inflammatory therapy in human lymphedema	Human pilot studies	Early clinical feasibility	[74]
VEGF-C mRNA nanoparticles reversing experimental lymphedema	Animal/preclinical regenerative therapy	Preclinical proof-of-concept	[76]
Phase 1 Lymfactin VEGF-C gene therapy + lymph node transfer	Phase 1 safety/early efficacy study	Early clinical feasibility	[77]
Anti-CTLA4 associated with reduced lymphedema risk	Retrospective melanoma cohort + mouse validation	Early clinical-translational feasibility	[78]
Online GEM program for BCRL	Protocol for pilot randomized controlled trial	Planned early clinical feasibility; no outcome evidence yet	[80]

## Data Availability

This article is a narrative review rather than a systematic review or meta-analysis. We searched PubMed, Web of Science, Scopus, and Google Scholar for English-language publications through April 2026 using combinations of the terms “cancer-related lymphedema”, “breast cancer-related lymphedema”, “secondary lymphedema”, “mechanobiology”, “mechanotransduction”, “tissue stiffness”, “interstitial fluid pressure”, “lymphatic contractility”, “elastography”, “ICG lymphography”, “bioimpedance”, “radiomics”, “artificial intelligence”, “machine learning”, “deep learning”, “compression”, “lymphatic surgery”, “VEGF-C”, “anti-fibrotic therapy”, and “digital twin”. We prioritized mechanistic studies, human clinical studies, consensus/guideline documents, externally validated prediction studies, and recent AI studies with explicit validation design. No datasets were generated or analyzed during the current study.
